# Older Age Results in Differential Gene Expression after Mild Traumatic Brain Injury and Is Linked to Imaging Differences at Acute Follow-up

**DOI:** 10.3389/fnagi.2016.00168

**Published:** 2016-07-13

**Authors:** Young-Eun Cho, Lawrence L. Latour, Hyungsuk Kim, L. Christine Turtzo, Anlys Olivera, Whitney S. Livingston, Dan Wang, Christiana Martin, Chen Lai, Ann Cashion, Jessica Gill

**Affiliations:** ^1^National Institute of Nursing Research, National Institutes of Health, BethesdaMD, USA; ^2^National Institute of Neurological Disorders, National Institutes of Health, BethesdaMD, USA

**Keywords:** traumatic brain injury, aging, inflammation, gene expression, imaging

## Abstract

Older age consistently relates to a lesser ability to fully recover from a traumatic brain injury (TBI); however, there is limited data to explicate the nature of age-related risks. This study was undertaken to determine the relationship of age on gene-activity following a TBI, and how this biomarker relates to changes in neuroimaging findings. A young group (between the ages of 19 and 35 years), and an old group (between the ages of 60 and 89 years) were compared on global gene-activity within 48 h following a TBI, and then at follow-up within 1-week. At each time-point, gene expression profiles, and imaging findings from both magnetic resonance imaging (MRI) and computed tomography were obtained and compared. The young group was found to have greater gene expression of inflammatory regulatory genes at 48 h and 1-week in genes such as basic leucine zipper transcription factor 2 (*BACH2*), leucine-rich repeat neuronal 3 (*LRRN3*), and lymphoid enhancer-binding factor 1 (*LEF1*) compared to the old group. In the old group, there was increased activity in genes within S100 family, including calcium binding protein P (*S100P*) and S100 calcium binding protein A8 (*S100A8*), which previous studies have linked to poor recovery from TBI. The old group also had reduced activity of the noggin (*NOG*) gene, which is a member of the transforming growth factor-β superfamily and is linked to neurorecovery and neuroregeneration compared to the young group. We link these gene expression findings that were validated to neuroimaging, reporting that in the old group with a MRI finding of TBI-related damage, there was a lesser likelihood to then have a negative MRI finding at follow-up compared to the young group. Together, these data indicate that age impacts gene activity following a TBI, and suggest that this differential activity related to immune regulation and neurorecovery contributes to a lesser likelihood of neuronal recovery in older patients as indicated through neuroimaging.

## Introduction

Traumatic brain injuries (TBIs) occur in about 5% of individuals over 60 years of age, and place them at a far greater risk for morbidity and mortality following TBI compared to younger cohorts (Ghorbani et al., 2014). In both the general population, and in older individuals, most TBIs are mild in severity and result in short-lasting symptoms (Centers for Disease Control and Prevention [CDC], National Center for Injury Prevention and Control, 2003, 2014); however, approximately 10% of mild TBI subjects do not fully recover (Carroll et al., 2004; Kashluba et al., 2008). Part of the heterogeneity in recovery from TBI is related to age, with older subjects being consistently at higher risk for poor or partial recovery (McIntyre et al., 2013; Sherer et al., 2015). Hukkelhoven et al. (2003) reported that the odds for a poor outcome following TBI increases by 40–50% per every 10 years of age, with those patients who are 60 years or older being at four times greater risk to not fully recover compared to a 30-year-old TBI patient. The age-related risk observed by Hukkelhoven et al. (2003) relates to insufficient recovery processes, as opposed to injury severity or type of injury. This illustrates that age-related factors contribute to poor recovery, and that a better understanding of the mechanisms of age-related compromise may inform the development of therapeutic interventions to mitigate age-related risk. In further support of this link, adults above 60 years of age with similar severity and type of injury more often require neurosurgical intervention following a moderate or severe TBI (Tierney et al., 2015). Following a mild TBI, being over 60 is the only independent predictor of insufficient recovery (Tierney et al., 2015). It is therefore important to develop a greater understanding of the pathophysiological processes following TBI which will ultimately improve diagnostics and interventions to reduce this age-related risk for older patients who sustained a TBI (Mellergard et al., 2012).

The higher morbidity and mortality rates in older TBI patients strongly suggest that age impacts neurological recovery following a TBI (Adekoya et al., 2002). TBIs place the brain at risk for accelerated aging, with the brains of TBI subjects appearing 5 years older than controls (Cole et al., 2015). Further, older patients who sustain a TBI are approximately 40% more likely to develop neurodegenerative disorders such as Parkinson’s disease (Gardner et al., 2015) and dementia (Gardner et al., 2014), a risk that is not observed in younger subjects. Together these studies illustrate that the consequences of TBI differ between the old and the young, suggesting the presence of distinct biological mechanisms underlying age-related differences in the response to TBI, that currently are poorly understood.

Aging affects a plethora of pathways important in the response to brain injury. Studies show that older patients with a TBI have greater alterations in blood–brain barrier (BBB) permeability, which may contribute to tissue damage after TBI (Farrall and Wardlaw, 2009) or stroke (Zeevi et al., 2010). Neuroinflammatory processes also differ between the young and old (Ritzel et al., 2015), with preclinical models consistently linking poor recovery to advanced age, a relationship mediated in part through greater neuroinflammation (Kumar et al., 2013; Webster et al., 2015; Gupta and Prasad, 2016). Furthermore, systemic immune system activity, drug metabolism and neuroendocrine processes are attenuated by the aging process (Gruver et al., 2007; Cekic and Stein, 2010) and likely contribute to insufficient recovery from brain injury in older individuals. Therefore we postulate that these age-related differences can be determined by comparing young and old patients who sustain a TBI through global gene-expression differences. We expect this line of research to provide a better understanding of the age-related molecular mechanisms that contribute to poor recovery in older TBI patients, and to ultimately lead to the identification or development of targeted therapeutic interventions to mitigate age-related TBI impairments.

Gene expression profiles provide an opportunity to under stand how complex biological systems relate to TBI (Michael et al., 2005; Staffa et al., 2012; Heinzelmann et al., 2014). Both preclinical and clinical studies have reported significant changes in gene expression following a TBI (Lu et al., 2004), however, a clear biomarker of this injury has yet to be determined (Barr et al., 2011). A plausible reason for this is that whereas preclinical studies are able to examine neuronal gene-activity changes clinical studies are restricted to the used of primarily peripheral blood. An example of this is a recent study in a rat model of TBI which showed that brain tissue-related changes in gene-activity were related to cell death and survival gene pathways (White et al., 2016), as well as inflammation gene-pathways (Sabir et al., 2015). Within preclinical studies, there are both studies using *in vitro* and *in vivo* induced TBI models, with a very interesting study that compared both models using a genome-wide approach to have differential gene expression related to genes that direct the function of amyloid precursor protein to the recycling pathway by direct binding and away from amyloid beta producing enzymes, in both TBI models (Lamprecht et al., 2016). Preclinical studies also highlight the ability of these models to use of peripheral gene expression in the pathophysiological processes following TBI (Hernandez-Ontiveros et al., 2013; Woodcock and Morganti-Kossmann, 2013). Therefore, these preclinical models provide invaluable insights into TBI-related gene-activity changes that cannot be determined in clinical studies.

In clinical studies, the examination of peripheral gene expression is supported by research demonstrating that central nervous system microglia communicate with peripheral immune cells, and that this interaction results in alterations in peripheral immune cell gene expression that can be detected in venous blood following TBIs (Raghavendra Rao et al., 2003; Woodcock and Morganti-Kossmann, 2013). Moreover, peripheral immune cells have access to, and are actively recruited to the meninges and parenchyma to participate in the protection and repair of neuronal and supporting cells and functional recovery (Schwartz et al., 2013). Evidence from our group (Heinzelmann et al., 2014) suggests that there are clinically relevant differences in gene expression in peripheral immune cells in TBI patients compared to controls. Other clinical studies also report down regulation of olfactory receptor genes in the peripheral blood following a TBI, which was linked to abnormal tau phosphorylation (Zhao et al., 2013); however, this study only examined gene expression at one time-point. Therefore, previous studies suggest that peripheral blood gene expression changes relate to TBIs. In the only known study to examine the impact of age in relation to TBI recovery and role of genetic predisposition, polymorphisms in brain-derived neurotrophic factor interacted with age following severe TBI and related to mortality risk (Failla et al., 2015); however, gene-function was not determined. These studies did not explicate the association between gene expression and tissue recovery evidenced in imaging scans, and even fewer studies compare processes in young and old patients, thereby limiting our ability to develop improved diagnostics, therapeutic screenings, and interventions for older patients with TBI (Mellergard et al., 2012).

In the present study, we describe the impact of age on recovery and biomarkers related to this by gene expression changes following TBI. We examined gene expression from peripheral blood during the acute period (within 48 h) and at follow-up (1-week), and examined age by comparing two groups, a young group (19–35 years old) and an old group (60–89 years old). We also compared the presence or absence of TBI-related magnetic resonance imaging (MRI) findings over time in both age groups. Possible links between gene expression and imaging findings may provide insights into which genes may serve as therapeutic target to promote recovery in older TBI patients.

## Materials and Methods

### Subjects

The Center for Neuroscience and Regenerative Medicine Traumatic Head Injury Neuroimaging Classification protocol (NCT01132937) enrolled subjects who had sustained a head injury within 48 h prior to presentation at either MedStar Washington Hospital Center (WHC), Washington, DC, USA or Johns Hopkins Suburban Hospital (SH), Bethesda, MD, USA. The protocol was approved by the National Institutes of Health Intramural Institutional Review Board, and all research was conducted in accordance with the committee’s recommendations. Prior to study procedures, written informed consent was obtained from all subjects. In order to test the difference by age, only two groups were compared; the young (19–35 years old, *n* = 33) and the old groups (60–89 years old, *n* = 33), while the intermediate age group (36–59 years old) was not analyzed further in this study to avoid the potential confounder of fluctuating levels of sex steroid hormones during the perimenopausal stage.

### Imaging

A conventional non-contrast computed tomography (CT) scan was obtained for clinical purposes. Following consent, a research MRI was obtained. MRI was conducted at two time points; within 48 h and 1-week post-injury. A standardized MRI protocol of approximately 25 min in duration was used which included: diffusion-weighted imaging (DWI), two T2^∗^-weighted sequences, pre- and post-contrast fluid-attenuated inversion recovery, 3D high resolution T1, and dynamic susceptibility contrast perfusion-weighted imaging. Depending on local site policy, subjects were administered one dose of a gadolinium based contrast agent, either 0.1 mmol/kg gadopentetate dimeglumine (Bayer HealthCare, Leverkusen, Germany) or gadobenate dimeglumine (Bracco Diagnostics, Monroe Township, NJ, USA). A power injector at 5 ml/s through a 22-18 gauge needle administered contrast to the antecubital vein. Images were obtained from 1.5 T (GE Medical Systems, Milwaukee, WI, USA) at SH and a 3 T (Philips, Cleveland, OH, USA) at WHC.

### Clinical Symptoms

Injury severity at initial presentation was assessed using the Glasgow Coma Scale (GCS; Teasdale and Jennett, 1974). The GCS is scored between 3 (worst) and 15 (no impairment), based on a patient’s eye response, verbal response, and motor response. The representative score is the total GCS score (Teasdale and Jennett, 1974). The severity of each symptom on the Neurobehavioral Symptom Inventory (NSI) was also measured using a 5-item scale [0 (none) to 4 (very severe)] that asks subjects to indicate the extent to which each symptom has disturbed them (Vanderploeg et al., 2015). These scores were obtained twice, at baseline within 48 h of injury and 1-week post-injury. The total score is the sum of cluster scores (vestibular, somatic sensory, cognitive, and affective). Total scores can range from 0 to 88, with 88 being the most severe (Vanderploeg et al., 2015).

### Microarray

Peripheral blood samples were collected in PAXgene blood RNA tubes at 48 h and 1-week post-injury. PAXgene tubes were incubated at room temperature for 2 h, at –20°C overnight, and then at –80°C until further processing. Total RNA was isolated using the PAXgene blood RNA kit (PreAnalytiX, QIAGEN, Venlo, Limburg, Netherlands) according to the manufacturer’s protocol. Using the GeneChip 3′ IVT Plus Expression kit (Affymetrix, Santa Clara, CA, USA), each RNA sample (100 ng) was reverse transcribed, converted to biotinylated cRNA, and hybridized to Affymetrix Human Genome U133 Plus 2.0 microarrays (Affymetrix, Santa Clara, CA, USA).

### Quantitative Real-Time PCR

Quantitative real-time PCR was performed with five genes selected from significantly upregulated genes in the young group at 48 h post-injury. Three genes, leucine-rich repeat neuronal 3 (LRRN3), POU2AF1, and noggin (NOG) were among top 5 upregulated genes and BACH2, PITPNC1 were randomly chosen. Two reference genes, TRAP1 and DECR1 were used to normalize data (Stamova et al., 2009). One microgram of total RNA of each subject was reverse transcribed using High-Capacity cDNA reverse transcription kits (Applied Biosystems, Foster City, CA, USA). Quantitative real-time PCR was performed with TaqMan^®^ probes in QuantStudio^TM^ 6 Flex Real-Time PCR system (Applied Biosystems, Foster City, CA, USA) with a total volume of 5 μl. PCR assay was performed in triplicate under the following condition based on the manufacture’s protocol; one cycle of 2 min at 50°C; 10 min at 95°C; and 40 cycles of 15 s at 95°C, and 1 min at 60°C. The instrumental software was used to get normalized threshold cycle (ΔCt) values, which were obtained from each subject by subtracting Ct of reference genes from Ct of target genes.

### Statistical Analysis

Microarray data were analyzed with Partek Genomics Suite version 6.6 (Partek Inc., St. Louis, MO, USA). We used the standard gene expression workflow in the Partek Genomics Suite. Gene expression was normalized by using “Standardize” option, which shifts genes to mean of 0 and scale to standard deviation of 1. For differential expression, we used ANOVA. We considered the age as the ANOVA factor and added contrast to get the fold change and ratio between the phenotype and control. Multiple testing correction was performed on the *p*-values to get the false discovery rates (FDR). We chose the genes with fold change greater than 1.5 and with the FDR smaller than 0.05. QIAGEN’s Ingenuity Pathway Analysis (IPA, QIAGEN, Venlo, Limburg, Netherlands) was used to determine which diseases and disorders related to observed gene expression changes, as well as top-gene networks implicated in these gene expression changes. Two-tailed chi-square tests were used in demographic features such as sex, race, ethnicity, frequency/duration of loss of consciousness (LOC)/post-traumatic amnesia (PTA), and injury mechanism to investigate the differences between age groups using SPSS version 22.0 (SPSS Inc., Chicago, IL, USA). The number of subject whose GCS is less than 15 was compared between age groups with two-tailed chi-square tests. Total NSI score from each two time points was compared between age groups with Mann–Whitney test. Differences were considered to be statistically significant when *p* < 0.05.

## Results

A total of 66 subjects were included in this study, of which 48 subjects had follow-up data at 1-week post-TBI. The demographic and clinical features of the total group (*n* = 66) as well as the young (*n* = 33) and old groups (*n* = 33) are described in **Table 1**. The majority of subjects were males (69.7%), and Caucasians (89.4%) with more than half experiencing LOC and PTA. There were no statistical differences in gender or race between groups. However, the young group experienced significantly more LOC (*p* = 0.006) and PTA (*p* = 0.012) than the old group. The most common mechanism of TBI was direct impact related to falls and motor vehicle accidents for both young and old groups.

**Table 1:** Demographic and clinical characteristics of the young and the old groups at 48 h and 1-week post-injury.

Variable	Young group	Old group	*p*-value
No.	33	33	
Male, *n* (%)	26 (78.8)	20 (60.6)	0.180
Age, median (IQR)	27.6 (23–32)	67.5 (64–80)	<0.001
Race, *n* (%)			
Caucasian	28 (84.8)	31 (93.9)	0.230
Latino/Hispanic	11 (33.33)	2 (6.06)	0.005
Post-traumatic amnesia, *n* (%)	25 (75.8)	15 (45.5)	0.012
Post-traumatic amnesia duration, *n* (%)			
1 s to 10 min	10 (40.00)	4 (26.67)	0.247
10 min to 30 min	4 (16.00)	5 (33.33)	
30 min to 1 h	5 (20.00)	0 (0.00)	
1 h to 12 h	3 (12.00%)	2 (13.33)	
12 h to 24 h	1 (4.00)	0 (0.00)	
Unknown	1 (4.00)	3 (20.00)	
Loss of consciousness, *n* (%)	27 (81.8)	14 (42.4)	0.006
Loss of consciousness duration, *n* (%)			
<1 min	4 (14.81)	1 (5.26)	0.155
1 min to 29 min	16 (59.26)	8 (42.11)	
30 min to 59 min	0 (0.00)	0 (0.00)	
1 h to 24 h	1 (3.70)	0 (0.00)	
Unknown	6 (22.22)	10 (52.63)	
GCS 48 h while admitted <15, *n* (%)	7 (21.2)	10 (30.3)	0.677
NSI total 48 h, median (IQR)	15.0 (11–26)	10.0 (4–28)	0.004
NSI total 1 week, median (IQR)	9.0 (4–19)	8.0 (1–14)	0.136
Injury mechanism, *n* (%)			
Acceleration/deceleration	5 (15.15)	1 (3.03)	0.551
Direct impact (blow to head)	7 (21.21)	9 (27.27)	
Direct impact (head against object)	8 (24.24)	9 (27.27)	
Fall (ground floor)	7 (21.21)	8 (24.24)	
Fall (height > 1 m)	6 (21.21)	6 (18.18)	

*IQR, interquartile range.*

The median score of GCS was between 14 and 15 (mild TBI) at each time point for both groups. Most of the subjects were mild TBI (GCS 14–15), and their interquartile range were 15. Therefore, we compared subjects whose GCS was less than 15 from each group, which was not significantly different between groups. The distribution of those with 15 was not different between these groups at any time-point. Self-reported symptoms were obtained using the NSI at 48 h and 1-week post-TBI with sub-cluster scores and the total score. At both time points, all NSI scores were higher in the young group compared to the old group.

CT scan results indicated that the young group has significantly fewer TBI positive findings than the old group (9.38 vs. 54.55%, respectively, *p* < 0.001) within 48 h post-TBI (**Table 2**). In contrast, MRI showed that the majority of subjects in both groups (about 75%) had evidence of a TBI within 48 h of injury. At the 1-week follow-up, the number of subjects with a positive MRI for TBI-related findings was significantly decreased in the young group (from 72 to 32%; *p* = 0.002), but not in the old group (75 to 76%).

**Table 2:** CT and MRI data at 48 h and 1-week post-injury for both groups.

	48 h (*n* = 66)			1 week (*n* = 48)		
	Young group (*n* = 33)	Old group (*n* = 33)	*p*-value	Young group (*n* = 25)	Old group (*n* = 23)	*p*-value
CT						
TBI (+)	3/32 (9.38%)	18/33 (54.55%)	<0.001			
Intracerebral hemorrhage	1/32 (3.13%)	4/33 (12.12%)	0.174			
MRI						
TBI (+)	24/33 (72.72%)^#^	25/33 (75.76%)	0.778	8/25 (32.00%)^#^	16/21 (76.19%)	0.003
TBI (+) only at 48 h (=resolve)				13/24 (54.17%)	2/21 (9.52%)	0.002
TBI no change in 1-week				8/24 (33.33%)	15/21 (71.43%)	0.017
TBI (+) only at 1-week				3/24 (12.5%)	4/21 (19.05%)	0.545

*This table describes initial findings at 48 h, and changes in the MRI scans at 1 week. ^#^p < 0.05 between the 48 h and 1-week in the same group.*

**FIGURE 1:**
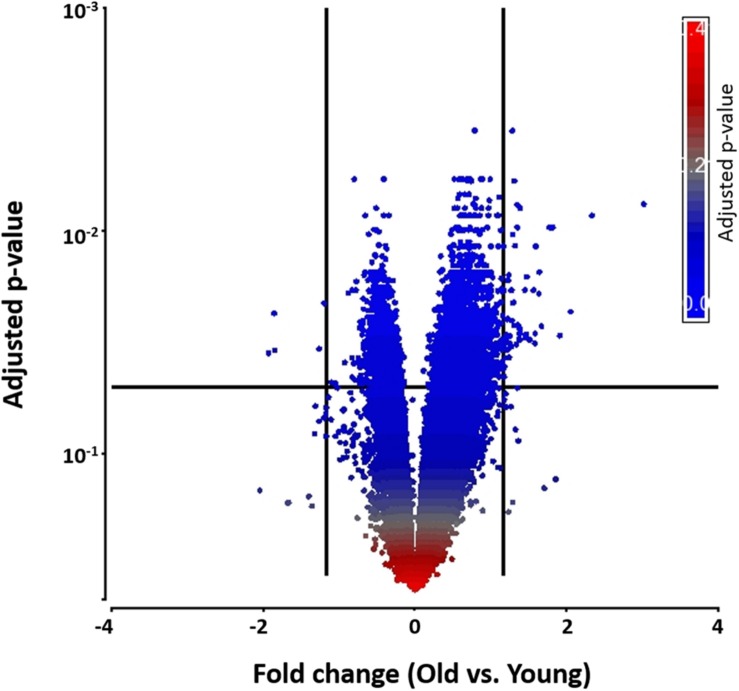
**A volcano plot that shows differentially expressed genes with statistical significance and fold change in the young and the old group.** Significant genes were selected by fold change (>1.5- or < –1.5-fold) and adjusted *p*-value (<0.05). Each dot denotes a gene. It was colored by adjusted *p*-value as shown in the legend. Fold change was evaluated as the ratio of the old to the young.

To begin exploring underlying pathways, we undertook a gene expression profiling analysis from peripheral whole blood at both 48 h and 1-week post-TBI and compared the young and old groups at both time-points. QC metrics of 66 subjects were shown in Supplementary Figure S1. Based on ANOVA result, the volcano plot displays significantly expressed genes against fold-change and adjusted *p*-value at 48 h post-TBI (**Figure 1**). At 48 h post-TBI, we found 56 transcripts annotated to 42 genes that were significantly upregulated in the young group, which include: LRRN3, NOG, and tumor necrosis factor receptor superfamily, member 17 (TNFRSF17; **Table 3**). **Table 4** shows classification of those genes based on their network functions. Genes associated with neurological and inflammation diseases were shown at **Table 5**. Five transcripts annotated to five genes were significantly upregulated in the old group including at 48 h post-TBI: S100 calcium binding protein P (S100P), and S100 calcium binding protein A8 (S100A8; **Table 6**). At 1-week post-TBI, we found 48 transcripts annotated to 28 genes that were significantly upregulated in the young group, including 19 genes that were also upregulated at the 48 h time period (67.86%; **Table 3**). In the old group, only one gene was significantly upregulated at 1-week, which was *BCAT1* (1.858 fold change). In order to verify the microarray result, quantitative real-time PCR was performed with five genes including LRRN3, POU2AF1, NOG, BACH2, and PITPNC1, which are upregulated in the young group at 48 h post-TBI and compared between young and old groups. Among five genes, the expression level of LRRN3, POU2AF1, and NOG was significantly higher in the young group, which correlates with microarray data (Supplementary Figure S2).

**Table 3:** Upregulated genes in the young compared to the old group at 48 h post-injury and its change at 1-week post-injury.

Symbol	Gene name	False discovery rate (*q*-value) at 48 h	Fold change at 48 h	False discovery rate (*q*-value) at 1-week	Fold change at 1-week
LRRN3	Leucine-rich repeat neuronal 3	0.008	2.849^∗^	0.005	3.692^∗^
IGHA1	Immunoglobulin heavy constant alpha 1	0.023	2.039^∗^	0.123	2.600^∗^
IGJ	Immunoglobulin J polypeptide, linker protein for immunoglobulin alpha and mu polypeptides	0.030	1.941^∗^	0.195	2.331^∗^
POU2AF1	POU class 2 associating factor 1	0.010	1.879^∗^	0.027	1.965^∗^
NOG	Noggin	0.010	1.852^∗^	0.005	2.234^∗^
IGLC1	Immunoglobulin lambda constant 1 (Mcg marker)	0.027	1.784^∗^	0.043	2.220^∗^
EBF1	Early B cell factor 1	0.015	1.767^∗^	0.141	1.592^∗^
PAX5	Paired box 5	0.019	1.763^∗^	0.098	1.770^∗^
NT5E	5′-nucleotidase, ecto (CD73)	0.012	1.736^∗^	0.021	1.656^∗^
IGHG1	Immunoglobulin heavy constant gamma 1 (G1m marker)	0.032	1.731^∗^	0.163	2.407^∗^
AFF3	AF4/FMR2 family, member 3	0.031	1.689^∗^	0.192	1.630^∗^
SF1	Splicing factor 1	0.016	1.667^∗^	0.363	1.532^∗^
TNFRSF17	Tumor necrosis factor (TNF) receptor superfamily, member 17	0.029	1.664^∗^	0.448	1.465
HIP1	Huntingtin interacting protein 1	0.030	1.656^∗^	0.552	1.378
ZBTB20	Zinc finger and BTB domain containing 20	0.008	1.619^∗^	0.122	1.523^∗^
IGHD	Immunoglobulin heavy constant delta	0.029	1.619^∗^	0.379	1.408
BACH2	BTB and CNC homology 1, basic leucine zipper transcription factor 2	0.028	1.616^∗^	0.019	1.849^∗^
BLNK	B cell linker	0.029	1.615^∗^	0.145	1.609^∗^
CAMK4	Calcium/calmodulin-dependent protein kinase IV	0.031	1.615^∗^	0.179	1.696^∗^
STRBP	Spermatid perinuclear RNA binding protein	0.010	1.598^∗^	0.021	1.584^∗^
IGKC	Immunoglobulin kappa constant	0.008	1.597^∗^	0.161	1.325
FCRL5	Fc receptor-like 5	0.032	1.589^∗^	0.514	1.333
SLC16A10	Solute carrier family 16 (aromatic amino acid transporter), member 10	0.012	1.581^∗^	0.076	1.479
PITPNC1	Phosphatidylinositol transfer protein, cytoplasmic 1	0.006	1.575^∗^	0.179	1.611^∗^
TSPAN13	Tetraspanin 13	0.027	1.575^∗^	0.146	1.541^∗^
KLHL24	Kelch-like family member 24	0.021	1.569^∗^	0.515	1.268
ETS1	v-ets avian erythroblastosis virus E26 oncogene homolog 1	0.021	1.565^∗^	0.296	1.359
AUTS2	Autism susceptibility candidate 2	0.010	1.564^∗^	0.057	1.726^∗^
CDCA7L	Cell division cycle associated 7-like	0.004	1.561^∗^	0.005	1.505^∗^
OSBPL10	Oxysterol binding protein-like 10	0.025	1.557^∗^	0.119	1.545^∗^
ABLIM1	Actin binding LIM protein 1	0.019	1.556^∗^	0.024	1.725^∗^
IQGAP1	IQ motif containing GTPase activating protein 1	0.030	1.543^∗^	0.697	1.281
P2RY10	Purinergic receptor P2Y, G-protein coupled, 10	0.032	1.541^∗^	0.077	1.617^∗^
LEF1	Lymphoid enhancer-binding factor 1	0.037	1.539^∗^	0.046	1.896^∗^
ERAP1	Endoplasmic reticulum aminopeptidase 1	0.017	1.531^∗^	0.383	1.455
HSP90AB1	Heat shock protein 90 kDa alpha (cytosolic), class B member 1	0.025	1.525^∗^	0.321	1.554^∗^
AGMAT	Agmatineureohydrolase (agmatinase)	0.029	1.519^∗^	0.018	1.724^∗^
MS4A1	Membrane-spanning 4-domains, subfamily A, member 1	0.037	1.517^∗^	0.193	1.645^∗^
WLS	wntless Wnt ligand secretion mediator	0.031	1.514^∗^	0.590	1.373
TPD52	Tumor protein D52	0.032	1.510^∗^	0.266	1.392
TCL1A	T cell leukemia/lymphoma 1A	0.041	1.507^∗^	0.411	1.476
IGHM	Immunoglobulin heavy constant mu	0.019	1.503^∗^	0.064	1.548^∗^
SGK223	Homolog of rat pragma of Rnd2	0.028	1.419	0.005	1.671^∗^
FAIM3	Fas apoptotic inhibitory molecule 3	0.040	1.363	0.021	1.665^∗^
AQP3	Aquaporin 3 (gill blood group)	0.045	1.470	0.034	1.658^∗^
TRAF5	TNF receptor-associated factor 5	0.049	1.416	0.043	1.642^∗^
PLXDC1	Plexin domain containing 1	0.029	1.327	0.001	1.595^∗^
MAN1C1	Mannosidase, alpha, class 1C, member 1	0.054	1.322	0.033	1.561^∗^
SCML1	Sex comb on midleg-like 1 (Drosophila)	0.019	1.460	0.020	1.527^∗^
ZNF827	Zinc finger protein 827	0.052	1.322	0.031	1.513^∗^
TCF4	Transcription factor 4	0.029	1.415	0.043	1.502^∗^

*Significance level was set at 1.5-fold change. ^∗^Identifies genes with a significant fold change.*

**Table 4:** Functional classification of significantly upregulated genes in young group at 48 h post-injury.

Functions	Genes
Cell-to-cell signaling and interaction, cellular development, cellular growth and proliferation (score = 27)	BACH2, CAMK4, CDCA7L, ERAP1, HIP1, IGHM, IQGAP1, KLHL24, LEF1, MS4A1, NT5E, PAX5, SF1
Cancer, cell death and survival, organismal injury and abnormalities (score = 20)	AFF3, BLNK, ETS1, FCRL5, HSP90AB1, PITPNC1, SLC16A10, TCL1A, TNFRSF17, TPD52, ZBTB20
Cellular development, cellular growth and proliferation, hematological system development and function (score = 15)	ABLIM1, AGMAT, AUTS2, IGHG1, IGLC1, LRRN3, NOG, POU2AF1, TSPAN13
Developmental disorder, hematological disease, hereditary disorder (score = 2)	P2RY10
Cell-to-cell signaling and interaction, cellular compromise, cellular function and maintenance (score = 2)	IGHA1

**Table 5:** Genes associated with neurological and inflammatory disorders in young group at 48h post-injury.

Diseases	*p*-value	Genes
Neurological diseases	2.48E-02–1.20E-05	ABLIM1, AUTS2, ERAP1, PITPNC1, TSPAN13, CDCA7L, IGHG1, IGHM, PITPNC1, MS4A1
Inflammatory response	3.66E-02–3.25E-05	BLNK, PAX5, POU2AF1, IGHA1, IGHM, MS41, IGHG1, NT5E, TCL1A, IGHM, MS4A1

**Table 6:** Upregulated genes in the old compared to the young group at 48 h post-injury and its change at 1-week post-injury.

Symbol	Gene name	False discovery rate (*q*-value) at 48 h	Fold change at 48 h	False discovery rate (*q*-value) at 1-week	Fold change at 1-week
S100P	S100 calcium binding protein P	0.0354	1.954	0.575	1.361
CA1	Carbonic anhydrase I	0.0235	1.905	0.361	1.630
ITGB2	Integrin, beta 2 (complement component 3 receptor 3 and 4 subunit)	0.0344	1.898	0.583	1.480
RNASE2	Ribonuclease, RNase A family, 2 (liver, eosinophil-derived neurotoxin)	0.0339	1.549	0.276	2.251
S100A8	S100 calcium binding protein A8	0.0212	1.515	0.230	2.469

*Significance level was set at 1.5-fold change.*

## Discussion

The higher morbidity and mortality rates consistently reported in older TBI patients strongly suggest that age impacts the neurological recovery response to TBI (Adekoya et al., 2002). In this study, for the first time we report gene expression differences in the young group (19–35 years old) and the old group (60–89 years old), with the young group having greater regulation of inflammatory activity, whereas the old group had more activity in S100 genes, which have previously been linked to TBIs. In addition to these gene expression differences, we also report that the young group had relatively improved neuronal recovery, as indicated by more subjects having no MRI evidence of TBI at follow-up compared to older subjects, despite similar MRI findings at 48 h following a TBI. Further, we used an alternative method, quantitative real time PCR to validate the gene expression differences determined through microarrays. These imaging findings suggest differences in neuronal recovery; however, the younger subjects endorsed far more TBI-related symptoms at both time-points. To the best of our knowledge, this is the first report to characterize the influence of aging on gene expression activity and its relationship to neuronal recovery by pairing biomarkers to imaging findings. These results suggest that there is a biological correlate for the long-established clinical observation of impaired recovery from neurological injury from TBIs in older TBI patients.

In this study, we report changes in several immune markers that suggest a down-regulation of B cell activities in the old TBI group. In our IPA, we identify 17 genes that are downregulated and one gene upregulated in the old group that are associated with the humoral immune response including immunoglobulin heavy constant alpha 1 (IGHA1), immunoglobulin lambda constant 1 (Mcg marker; IGLC1), and immunoglobulin J polypeptide, linker protein for immunoglobulin alpha and mu polypeptides (IGJ). The downregulation of these genes and the upregulation of ITGB2 suggest that there may be an immunosuppressive response that could translate to a decrease in the number of B lymphocytes and quantity of immunoglobulin. The role of B lymphocytes in TBI has not been established in the literature, but some of the genes downregulated in the old group could be involved in injury resolution. For example, BTB and CNC homology 1 (BACH2) decreased in expression in the old group from the acute point to follow-up point (–1.616 to –1.849, respectively) compared to the young group. BACH2 is a coding gene for basic leucine zipper transcription factor 2, which is expressed in B cells. In humans, genome-wide association studies link polymorphisms in the BACH2 locus to autoimmune and inflammatory conditions (Polychronakos and Li, 2011; International Multiple Sclerosis Genetics Consortium et al., 2013). Preclinical models link BACH2 to regulation of CD4+ T cells, and show that it is protective of initiating excessive inflammation (Roychoudhuri et al., 2013). Moreover, in preclinical models of ischemic injury, B cell deficiency was found to exacerbate histological damage and functional outcomes, while adoptive transfer of B cells was shown to decrease infarct size and improve neurological deficits (Li et al., 2013). Gene expression is also related to inflammatory regulation shown in a preclinical model of a mild single TBIs within the hippocampus (Tweedie et al., 2016). If indeed the gene expression profile we detected promotes a suppression of B lymphocyte function, then this could be a mechanism for inhibiting the neuroprotective effects of the humoral immune response through T cell regulation and secretion of interleukin 10, thereby contributing to worse outcomes reported in older adults with a TBI. Future studies are needed to examine the role of B cell-related genes in the context of TBI and how it relates to clinical outcomes, and contributes to age-related differences in TBI recovery.

Within the old group we also report genes within the S100 family to be significantly upregulated at 48 h post-injury, including S100P and S100A8. Human S100 encoding gene contains 25 family members, which help regulate intracellular levels of calcium (Zimmer et al., 2005), and is essential in neuronal recovery from injury in clinical samples. Specifically, S100B protein elevations are linked to poor prognosis following a TBI, suggesting that gene-activity related to this neuronal pathology following (Di Battista et al., 2015). S100P mediates cell proliferation by binding the receptor for advanced glycation end products to activate signaling pathways including extracellular signal-regulated kinase and NF-κB. S100A8 is expressed in activated macrophages and microglial cells (Beschorner et al., 2000). S100A8+ microglia are significantly increased after severe TBI in tissue studies (Beschorner et al., 2000; Engel et al., 2000), with those who were 69–99 years old having significantly more activity compared to subjects 20–59 years old (Cribbs et al., 2012). In the present study, *S100A8* was significantly increased both at 48 h and 1-week. Our findings, within the context of previous studies, suggest that upregulated *S100A8* contributes to the differential response of the older subjects in recovery from TBIs.

We also report that the inflammatory regulating genes like LRRN3 and lymphoid enhancer-binding factor 1 (*LEF1*) were highly upregulated at both time points in the young group compared to the old group. Both of these genes are related to T cell function and thought to be a part of immunosenescence (Cao et al., 2010; Remondini et al., 2010). Expression of these genes is reduced across studies of older individuals (Hong et al., 2008; Harries et al., 2011), suggesting that older subjects are not able to regulate inflammation as well as younger subjects can, which may contribute to a greater burden of immune disorders within older individuals, and a lesser ability to recover from TBI. In support of this, in a preclinical model of mild TBI, gene activity related to inflammation was increased in the hippocampus following injury, and were related to neuronal pathology, yet the impact of age was not determined (Sabir et al., 2015). Therefore, additional studies to understand how age impacts inflammatory activities in both clinical and preclinical studies are needed to explicate the role of inflammation in neuronal pathology.

After a TBI, breakdown of the BBB facilitates the passage of inflammatory molecules and cells into a previously immune protected environment (Das et al., 2012). Immune activities following TBI remain poorly understood in clinical studies. In preclinical studies we understand that coordination of the neuroinflammatory response to TBI are essential to clear damaged tissue and permit recovery; however, an excessive inflammatory response, as seen in severe TBI, can be neurotoxic (Kumar and Loane, 2012). Thus, it is a balance of immune activities at the correct time points following TBI that are critical, but not yet well described in clinical models of more mild TBIs (Ziebell and Morganti-Kossmann, 2010; Corps et al., 2015). Therefore, our finding that regulation of immune activities at 48 h following a TBI relates to a greater rate of MRI resolution in young TBI patients, provides initial evidence of how immune activities regulate this complex response.

Lastly, we found that NOG expression was also significantly decreased in the old group from 48 h to 1-week post-injury (–1.852 to –2.234 fold change). NOG is a protein coding gene for noggin, an extracellular bone morphogenetic protein (BMP) antagonist. BMP is a member of the transforming growth factor-β superfamily that increases in cerebrospinal fluid (CSF) within the first days after TBI from severe TBI patients (Morganti-Kossmann et al., 1999), and is associated with neuronal repair and neuroregeneration. Although the role of noggin as it relates to brain injury is not well described in the clinical literature, preclinical studies show that inhibition of BMP by noggin promotes neurological recovery from ischemic brain injury (Samanta et al., 2010) and recovery from intraventricular hemorrhage (Dummula et al., 2011). Together, these preclinical studies suggest that increased activity of noggin in the brain enhances recovery from injury through microglial activation and oligodendrogenesis (Bragdon et al., 2011). Moreover, BMP (Yousef et al., 2015) and NOG (Bonaguidi et al., 2008) signaling changes with age and relates to age-related neurological impairments and reductions in neuroregeneration (Yousef et al., 2015), providing some insights into our observation that this increase in gene activity is unique to the younger TBI cohort. By linking peripheral NOG and BMP gene-activity to resolution of TBI-related imaging findings, we suggest that gene activity contributes to the age disparity in neurological recovery from TBI, because younger subjects are more likely to initiate gene-activity changes that promote TBI recovery.

MRI can detect signs of injury including micro-hemorrhage, small areas of contusion, or gliosis that may not be observable by CT. MRI findings are linked to recovery at 3 months in mild TBI patients (Yuh et al., 2013). We also report that in the young group, there was a greater likelihood of having no image findings related to TBI at 1-week, compared to those in the old group. At baseline, the rates of MRI+ findings in these two groups were similar. Our findings support previous reports of greater neurological injury in older TBI patients, including larger lesion volumes on MRI, despite similar types and severity of TBIs (Schonberger et al., 2009), decreased mood and greater cognitive impairments, and higher overall disability after TBI (Marquez de la Plata et al., 2008).

Despite the similarities in GCS scores and mechanisms of injury with the young and old group, we found that the young group reported more symptoms of acute injury including PTA and neurological symptoms based on the NSI at both time-points. We found these results to be surprising, as the previous literature suggests that older patients are not necessarily under-endorsers of symptoms following a TBI (Hukkelhoven et al., 2003; Schonberger et al., 2009). It may be that aging occludes the changes in symptom severity or performance and leads to older patients not linking symptoms to the TBI, resulting in these measures of symptoms to not be as high in older patients. This finding can be addressed in future studies with a larger cohort and more objective measures.

This study is limited on comparing the difference in gene expression between young and old TBI patients, precluding age-matched healthy, non-TBI group. Including age-matched non-TBI subjects in the future study would provide baseline information of gene expression changes following TBI in each age group. This study was also limited by examining only old and young cohorts, thereby excluding the middle aged; however, in this initial study, this design provides for the first examination of the impact of age on TBI recovery and biological mechanisms. We acknowledge that the groups we used to define young and old needs to be better addressed in a more comprehensive fashion in future studies, yet we determined these groups based on current studies of aging, and feel that this is the initial step in examining the impact of age on biological functioning.

Our findings that age influences the response to TBI provides impetus for the consideration of age in the care of TBI patients, and justification for future studies to elucidate therapeutic targets that can be altered with pharmacological agents to promote recovery from TBI in older populations who are at higher risk. Furthermore, especially for the older group, immune-system supportive therapy such as immune-modulating nutritional supplements may improve the long-term outcome after TBI. These future studies will provide critical data to elucidate therapeutic targets that can be altered with pharmacological agents to promote recovery from TBI in older populations who are at higher risk.

## Author Contributions

Y-EC ran gene expression analysis and wrote gene expression part of the manuscript. LL organized the study that this paper collected data from, and he helped with imaging analysis and writing in the manuscript. HK processed and ran all gene expression samples in Affymetrix. LCT collected and organized clinical measures and imaging data. AO organized gene expression data and helped write the manuscript. WL created tables, formatted manuscript, and helped edit. DW and CM ran gene expression samples in Affymetrix and assisted in gene expression research and analysis. CL assisted in gene expression research and analysis. AC helped edit the paper, and provided support in organizing the study. JG wrote part of the manuscript, provided support in conceptualizing gene expression results, oversaw the entire project and provided genomics expert knowledge.

## Conflict of Interest Statement

The authors declare that the research was conducted in the absence of any commercial or financial relationships that could be construed as a potential conflict of interest.
