# Supplementary material for: Older Age Results in Differential Gene Expression after Mild Traumatic Brain Injury and Is Linked to Imaging Differences at Acute Follow-up
Source: Front Aging Neurosci. 2016 Jul 13;8:168. doi: 10.3389/fnagi.2016.00168 (PMC4942460; doi:10.3389/fnagi.2016.00168)
Supplement: Supplementary file 1 [file Image_1.PDF]

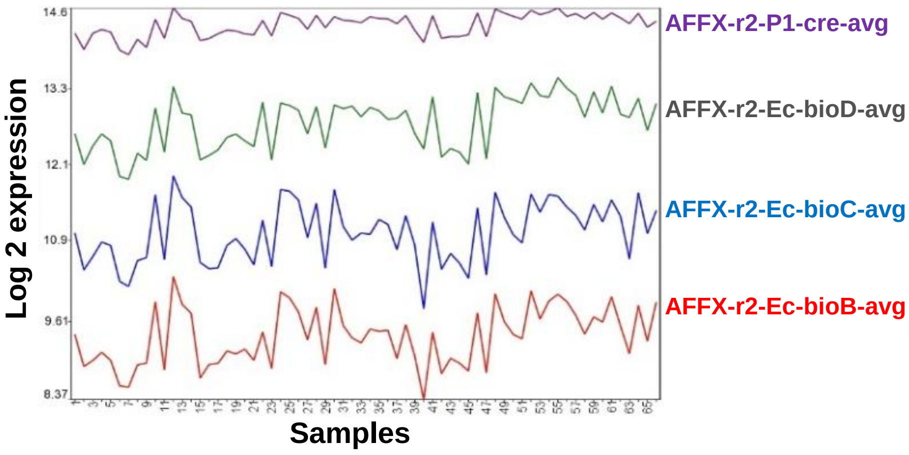

Figure S1. Log2 intensity of Affymetrix hybridization quality control probe-sets on 66 chips for the hybridization controls. The orders of the log2 expression are as expected, showing no anormaly.

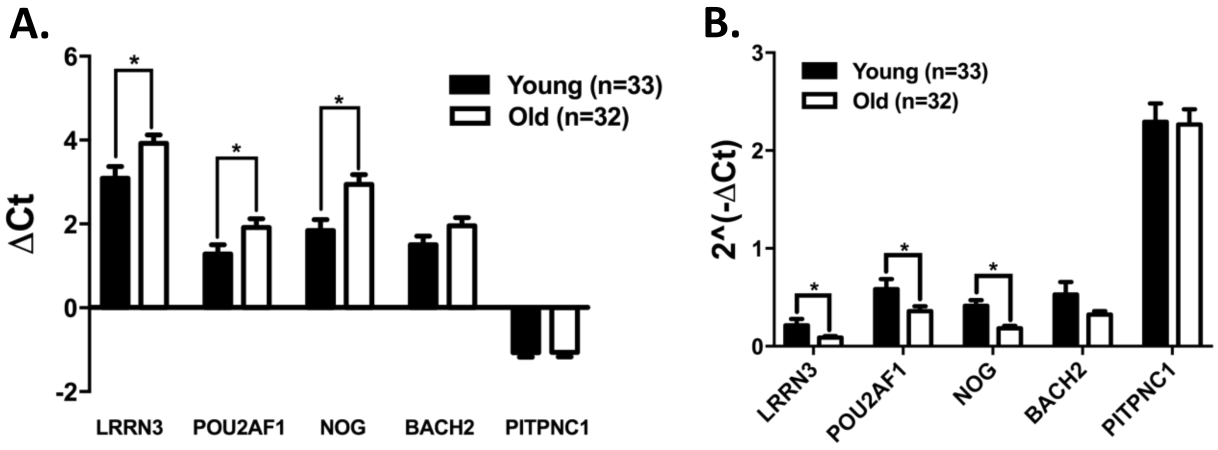

Figure S2. Expression of 5 genes, LRRN3, POU2AF1, NOG, BACH2 and PITPNC1, which were significantly increased in the young group at 48hrs post-injury in microarray was verified with quantitative RT-PCR. ΔCt values were obtained by normalizing to reference genes, TRAP1 and DECR1. Both ΔCt and 2^(-ΔCt) values of each gene were compared between young and old groups using Mann-Whitney test, showing that the expression level of LRRN3, POU2AF1, and NOG is significantly higher in the young group. Values are mean ± SE, *: $p<0.05$ between the young and old group.
